# Cold-hearted or cool-headed: physical coldness promotes utilitarian moral judgment

**DOI:** 10.3389/fpsyg.2014.01086

**Published:** 2014-10-02

**Authors:** Hiroko Nakamura, Yuichi Ito, Yoshiko Honma, Takuya Mori, Jun Kawaguchi

**Affiliations:** ^1^Department of Psychology, Graduate School of Environmental Studies, Nagoya UniversityNagoya, Japan; ^2^Department of Social and Human Science Informatics, School of Informatics and Sciences, Nagoya UniversityNagoya, Japan

**Keywords:** embodiment, moral dilemmas, coldness, empathy, construal level

## Abstract

In the current study, we examine the effect of physical coldness on personal moral dilemma judgment. Previous studies have indicated that utilitarian moral judgment—sacrificing a few people to achieve the greater good for others—was facilitated when: (1) participants suppressed an initial emotional response and deliberately thought about the utility of outcomes; (2) participants had a high-level construal mindset and focused on abstract goals (e.g., save many); or (3) there was a decreasing emotional response to sacrificing a few. In two experiments, we exposed participants to extreme cold or typical room temperature and then asked them to make personal moral dilemma judgments. The results of Experiment 1 indicated that coldness prompted utilitarian judgment, but the effect of coldness was independent from deliberate thought or abstract high-level construal mindset. As Experiment 2 revealed, coldness facilitated utilitarian judgment via reduced empathic feelings. Therefore, physical coldness did not affect the “cool-headed” deliberate process or the abstract high-level construal mindset. Rather, coldness biased people toward being “cold-hearted,” reduced empathetic concern, and facilitated utilitarian moral judgments.

## Introduction

Personal moral dilemma tasks examine whether it is permissible to sacrifice a few people for the sake of the greater good. For example, the footbridge dilemma scenario asks whether you would push an innocent person on a footbridge into the path of runway trolley and kill them in order to save five other lives (Thomson, 1976). Based on the situation, people may think of killing this one innocent person in one of two ways—as either “cold-hearted” or “cool-headed.” Studies of embodied cognition have revealed that such “cold-warm” expression is not just a metaphor, but temperature perception can affect social judgment (Williams and Bargh, 2008); thus, the present study tested the effect of temperature perception on moral dilemma judgment.

There are two common ways to make a moral dilemma judgment, and the dual process theory of moral judgment suggests that different processes are engaged in each judgment (e.g., Greene et al., 2004). Deontological judgments prefer the rights of the individual (e.g., “It's wrong to push the man”) and relate to intuitive and emotional process such as empathetic concern for a victim. In contrast, utilitarian judgments favor the greater good (e.g., “It's better to save the five”) and relate to a deliberate process of suppressing initial emotional responses and calculating the utility of the outcomes (Greene et al., 2001, 2008) showed that personal moral dilemmas activated the brain regions in which emotional responses occur (e.g., the medial frontal gyrus, posterior cingulate gyrus, and angular gyrus) and produced more deontological judgment than moral dilemma scenarios without salient emotional content. Greene et al. (2004) argued that there are conflict between evolutionally-old socio-emotional responses (deontological) and recently-evolved abstract reasoning (utilitarian) in personal moral dilemma task, and brain regions, which related with cognitive conflict (anterior cingulate cortex) and cognitive control (dorsolateral prefrontal cortex), were activated for answering utilitarian judgment in difficult personal moral dilemmas. Many studies also supported the relation between deliberate process and utilitarian judgment in high-conflict personal moral dilemmas; for example, cognitive load increased response times in utilitarian judgment (Greene et al., 2008), induced time pressure to answer quickly and intuitively increased deontological judgment (Suter and Hertwig, 2011), and a tendency toward a “need for cognition” was related to utilitarian judgment (Bartels, 2008).

Recent studies have shown that psychological distance and construal level also affect moral judgments. According to the construal level theory (Trope and Liberman, 2010), psychological distance is an egocentric representation that something is close or far away from the self, and psychological distance relates to levels of construal. A distant representation is linked to high-level construal, which is relatively abstract and focuses on distant goals. On the other hand, a close representation is linked to low-level construal, which is relatively concrete and focuses on the immediate means (Fujita et al., 2006). Eyal et al. (2008) argued that general moral rules (e.g., “it is wrong to steal,” “donating to charity is noble”) are related to high-level construal, whereas context-specific considerations are related to low-level construal.

In moral dilemma judgment, utilitarian judgments give priority to the ends (e.g., “to save many people”) and relate to high-level construal, whereas deontological judgments often give priority to the means (e.g., “to not push and kill an innocent man”) and relate to low-level construal. Aguilar et al. (2013) showed that psychologically distant event representations, such as temporally and spatially distant events, prompt utilitarian decisions. Amit and Greene (2012) reported that visual processing, which is more concrete than verbal processing, is positively related to deontological judgment. The relationship between construal level and the intuitive-deliberative process in moral judgment has been the source of controversy. Amit and Greene (2012) argued that construal level and dual processes were interrelated; i.e., the low-level construal features of the scenario (e.g., how to sacrifice the victim) triggered intuitive and emotional responses. On the other hand, Eyal et al. (2008) discussed the distinction between general and rule-based moral judgments (high level construal) vs. contextually based moral judgment (low level construal), which is not related to cognitive effort.

Several studies have indicated an alternative route to utilitarian judgment. In these findings, utilitarian judgments arise not only from a “*cool-headed*,” deliberate process or high-level construal mindset, but they also arise from a “*cold-hearted*” deficit of empathic concern and social emotion. For example, utilitarian judgment in a personal moral dilemma situation has been positively correlated with antisocial personality traits, such as psychopathy (Bartels and Pizarro, 2011) and psychoticism (Wiech et al., 2013), or with a reduction of “empathic concern” (Gleichgerrcht and Young, 2013). In addition, damage of ventromedial prefrontal cortex, which involves socially related emotions, produced more utilitarian judgment (Koenigs et al., 2007) and failed to generate a skin conductance response before making utilitarian judgment (Moretto et al., 2010). Wiech et al. (2013) suggested that reduced empathic concern and a reduction in emotional aversion to harming others facilitate utilitarian judgment without engaging deliberate processes.

Studies have also shown a relationship between moral judgment and embodied cognition. In a personal moral dilemma situation, scenarios with physical contact (Cushman et al., 2006) or personal force, in which the victims are sacrificed by the agent's muscle (e.g., by pushing), resulted in fewer utilitarian judgments (Greene et al., 2009). For example, in footbridge dilemmas, it was less acceptable to drop a victim by pushing him than by using a trap door and a remote switch. However, the effect of embodiment on moral dilemma judgment was not clear from the results of these studies.

Studies of embodiment cognition show that temperature perception influences interpersonal relationships. Williams and Bargh (2008) showed that, compared with physical coldness, physical warmth prompted positive impressions toward a target person and increased prosocial behavior. Physical warmth further prompted close social proximity toward the target person (IJzerman and Semin, 2009; Fay and Maner, 2012). Conversely, inducing social closeness leads people to perceive higher ambient temperatures (IJzerman and Semin, 2010), and social exclusion induces perceptions of physical coldness (Zhong and Leonardelli, 2008). Williams et al. (2009) explained the interrelations between bodily state and cognition on the basis of *scaffolding* processes. Scaffolding processes are refers to the processes which people integrate incoming information with extant knowledge structure, especially in early childhood. For example, the abstract concept of psychological distance (e.g., interpersonal proximity) is scaffolded upon the perceptual and body-based information (e.g., close physical contact, warm sensations). The link between physical warmth and social relation is said to be very basic, because social relations are learned through bodily contact and sensing others' body temperature in early in life (Williams and Bargh, 2008; Williams et al., 2009; IJzerman and Koole, 2011; Fay and Maner, 2012). For example, Bowlby (1969) suggested that an innate need for direct physical contact with a caretaker involves the experience of physical warmth. Harlow's (1958) study showed that young monkeys preferred to stay close to a warm cloth surrogate mother rather than a cold wire mother, and monkeys raised with the cold wire mother had trouble with social development.

The aim of the current study is to examine the effect of temperature perception on moral dilemma judgment. For this purpose, we exposed participants to either extreme cold or typical room temperature and then asked them to respond to personal moral dilemmas. We hypothesized two possible relationships between physical coldness and moral dilemma judgment. One is that cold temperature may affect moral judgment via inducing a high-level construal mindset. Construal level theory suggests that various psychological distances (e.g., spatial, temporal, and social) are interrelated and connected with levels of construal (Trope and Liberman, 2010). Physical coldness cues social distance from the target (IJzerman and Semin, 2009), and social distance connects with high-level construal mindsets; therefore, physical coldness may facilitate a focus on abstract goals and prompt utilitarian moral judgments. Furthermore, there is a possible relationship between construal level and dual processes in moral judgment (Amit and Greene, 2012); therefore, a high construal feature of the scenario may trigger deliberate and effortful thought. It is predicted that participants with a high-level construal mindset may take more time and thus make more utilitarian choices.

The other possible relationship is that of cold temperatures prompting utilitarian moral judgment via reducing empathic concerns toward sacrificing people. IJzerman and Koole (2011) pointed that physical warmth is relevant to communal sharing relationships in relational models theory (Haslam and Fiske, 1999; Fiske, 2004). Communal sharing relationships are altruistic relationships such as are experienced by families and close friends, and communal sharing relationships are often constructed through bonding experiences such as intimate touch, sex, and nursing. Rai and Fiske (2011) discussed the links between relational model and moral motives, and they said that communal sharing relationships are connected by the *Unity* moral motive, which is directed toward caring for in-groups through a sense of collective responsibility and common fate. The unity moral motive fosters caring for others in the group who are in need or those who have been harmed; thus, unity facilitates compassion and empathic emotions. If physical warmth and social proximity are the basis for communal sharing relationships and their corresponding moral motive of unity, coldness may decrease unity motives and their related emotions, such as empathy, compassion, and emotional aversion to harming others. Therefore, it is predicted that physical coldness may increase social distance and decrease empathic concern toward the sacrificed victim, thereby reducing the conflict between empathic-deontological judgment vs. utilitarian judgment. Consequently, coldness may facilitate utilitarian judgment without engaging deliberate thought and the high-level construal mindset.

## Experiment 1

Experiment 1 aimed to test the effect of coldness on high-conflict personal moral dilemma judgment and whether the effect of coldness is related to construal level and deliberate thought. According to the construal level theory, utilitarian judgment relates to abstract high-level construal features (Aguilar et al., 2013). If coldness fosters social distance and increases psychological distance, it may set participants in a higher-level construal mindset and facilitate utilitarian judgment. It was argued that construal level and dual processes were interrelated (Amit and Greene, 2012), and it is possible that high-level construal and the abstract feature of the scenario may trigger deliberate thought. Therefore, we predict that, compared with room temperature, participants in the colder temperature condition may show more high-level construal mindset, take more time, and make more utilitarian judgments for high-conflict personal moral dilemmas. In addition, as in previous studies, participants who are in a high-level construal mindset (Aguilar et al., 2013) or who take more time to answer the dilemmas (Suter and Hertwig, 2011) may show more utilitarian judgment in both temperature conditions.

In this experiment, we presented high-conflict personal moral scenarios as target stimuli, and we presented low-conflict personal moral scenarios as fillers. High-conflict personal moral scenarios evoked competition between deontological judgment and utilitarian judgment, whereas low-conflict personal moral scenarios lacked this degree of competition. It was showed that almost all the participants produced deontological judgment, and judgment latency was faster in the low-conflict scenario than in the high-conflict scenario (Koenigs et al., 2007). In addition, patients with lesions in vmPFC, a brain region necessary for the generation of social emotions, produced more utilitarian judgment in high-conflict moral dilemma situations than healthy controls, but both vmPFC patients and healthy controls produced deontological judgment in the low-conflict situation (Koenigs et al., 2007; Moretto et al., 2010). Therefore, all participants may endorse deontological judgment in low-conflict personal moral scenarios, and the effect of physical coldness may be observable only in high-conflict personal moral scenarios.

### Methods

#### Participants

This study was approved by the faculty ethics committee, and all participants provided informed consent. Forty-seven Japanese undergraduates (*M*_age_ = 19.37, *SD*_age_ = 1.25; 24 female, 23 male) participated in the study in exchange for a partial course credit. One participant was excluded for misunderstanding the instructions. Simmons et al. (2011) suggest that the experimenter must collect at least 20 observations per cell in order to have enough power to detect most effects; therefore, we gathered data from 23 participants for each condition.

#### Measures

***Moral judgment task***. The scenarios were randomly selected from a battery of 60 moral dilemmas developed by Greene et al. (2001). They were translated into Japanese and checked by an English-Japanese bilingual speaker. The target stimuli in the present study were 13 high-conflict personal moral dilemmas: Sacrifice, Crying Baby, Footbridge, Vaccine Test, Sophie's Choice, Lifeboat, Ecologists, Vitamins, Euthanasia, Lawrence of Arabia, Submarine, Bomb, and Preventing the Spread. We also presented eight low-conflict personal moral dilemmas to reduce repetition: Country Road, Plane Crash, Hired Rapist, Infanticide, Architect, Hard Times, Transplant, and Smother for dollars.

***Behavior identification form***. To measure construal level, we translated 25 items from the Behavior Identification Form (BIF; Vallacher and Wegner, 1989) into Japanese. The BIF presents 25 items about a target behavior (e.g., “locking a door”) and asks which one of two alternate descriptions the participants preferred. One described the behavior in terms of its concrete means (e.g., “putting a key in the lock”), and the other described it in terms of abstract outcome (e.g., “securing the house”). An overall score was obtained by adding the number of abstract high-level descriptions selected by a respondent across 25 behaviors. This score total was used as an index of level of action identification. Scores ranged from 0 to 25, with higher scores indicating stronger preferences for high-level action identifications. Thus, these scores were an index of a high-level construal mindset (Fujita et al., 2006).

***Positive and negative affect scales***. The 16-item Japanese version of Positive and Negative Affect Scales (PANAS) asks participants to report the extent to which, at the present moment, they feel the 8 positive (active, proud, strong, attentive, determined, interested, alert, enthusiastic) and 8 negative (scared, afraid, upset, nervous, distress, jittery, ashamed, irritable) emotions on a 6-point scale (1 = *Strongly disagree*, 6 = *Strongly agree*) (Tellegen et al., 1988; Sato and Yasuda, 2001).

### Procedure

A desktop computer with E-Prime software (Psychology Software Tools, 2002, Pittsburgh, PA) controlled the presentation of stimuli, timing operation, and data collection. Each participant entered a quiet room with a typical room temperature one by one, and they sat in front of a computer screen throughout the experiment. The experimenter also stayed in the room during the experiment for instructing and to confirm the participants' understanding of the task.

The current experiment was presented as two unrelated studies. One was ostensibly a “commercial product evaluation” study, and the second was a “judgment” study. Because present study asked more questions than previous studies, we modified temperature manipulation procedure of previous studies, which manipulated the temperature by holding a cup with warm or cold water (e.g., Williams and Bargh, 2008; IJzerman and Semin, 2009). In present study, participants were asked to keep wearing the scarf with frozen internal water packs or internal water packs at room temperature during the experiment.

Participants were asked to answer “commercial product evaluation” study. First, participants answered the PANAS: each emotion was presented on a single screen with the scales at the bottom, and there was no time limit. Then, participants were given a scarf with either frozen internal water packs (the cold condition) or internal water packs at room temperature (ordinary “room temperature” condition). The participants were instructed to put the scarf around their necks and to answer 10 questions about the scarf (e.g., “This scarf is the right size”) on a 7-point scale (1 = *Strongly disagree*, 7 = *Strongly agree*). The product evaluation section included one question about product temperature: “This scarf is cold.” Each product evaluation question was presented on a single screen with the scales at the bottom, and there was no time limit. After the product evaluation questions, they answered the second round of the PANAS. The participants were told that this product evaluation study also examined the prolonged use of the scarf. The experimenter asked them to keep wearing the scarf and to join a judgment study, which was not related to the product evaluation study.

In the judgment study, participants responded to 21 moral dilemma scenarios. Procedure of moral dilemma judgment was based on Moretto et al., (2010). Each dilemma was presented as text through a series of two screens. The first screen described the scenario and was presented for 45 s. The second screen posed a question, and the participants judged the moral acceptability of the proposed utilitarian action in each dilemma using a 7-point scale (1 = *completely inappropriate*, 7 = *completely appropriate*), and their response times were measured. There was no time limit. Participants were told to respond as soon as they had reached a decision. In the inter-trial interval, a blank screen was displayed for 1 s.

Following the moral dilemma task, participants completed the BIF task. Each target behavior was presented on a single screen with the two options at the bottom. There was no time limit. Participants then responded to the product evaluation questions again and answered 10 questions about the scarf. The final task was to complete the third round of the PANAS. After the experiment, the participants were thanked and debriefed. Participants were explained purpose of the experiment, and asked whether they were suspicious about the relation between product evaluation task (temperature manipulation) and judgment task. None of the participants reported being suspicious about the experimental purpose. Participants took about 50 min to complete the all tasks.

### Results

#### Manipulation check

Reaction times (RTs) of the moral judgment were natural log transformed before analysis, and mean RTs were obtained via inverse transformation. Table 1 shows the means and 95% confidence intervals of room temperature, coldness rating, BIF score, PANAS scores, and RTs both in the cold and ordinary room temperature conditions.

**Table 1:** **Means and 95% confidence intervals (CIs) of temperature, coldness rating, BIF score, PANAS scores, and RTs in cold and ordinary temperature conditions in Experiment 1**.

**Measures**		**Cold**			**Ordinary**		
		**Mean**	**95% CI**		**Mean**	**95% CI**	
Room temperature	(Celsius)	22.09	21.04	23.14	21.74	20.55	22.93
Coldness rating	1st	6.65	6.44	6.86	5.52	4.85	6.20
	2nd	6.22	5.78	6.65	4.30	3.53	5.08
BIF score		9.83	7.93	11.72	9.61	7.79	11.42
RTs (ms)	Low conflict	4248			4504		
	High conflict	5042			5580		
PANAS 1st	Positive	2.89	2.64	3.14	2.89	2.52	3.27
	Negative	2.34	1.98	2.71	2.26	1.89	2.64
PANAS 2nd	Positive	2.67	2.36	2.98	2.66	2.28	3.04
	Negative	2.20	1.84	2.56	2.03	1.67	2.39
PANAS 3rd	Positive	2.40	2.11	2.68	2.28	1.79	2.77
	Negative	2.32	1.86	2.77	2.20	1.84	2.56

There was no significant difference in room temperature between the cold and ordinary room temperature condition, *t*_(44)_ = 0.45, *p* = 0.652, *d* = 0.14. The scarf with frozen internal water packs was evaluated to be colder than the scarf with internal water packs at room temperature, both immediately after the participants started wearing it, *t*_(44)_ = 3.31, *p* = 0.002, *d* = 0.98, and after they finished the moral judgment task and BIF, *t*_(44)_ = 3.31, *p* = 0.002, *d* = 0.98.

#### Moral judgment

**Figure 1:**
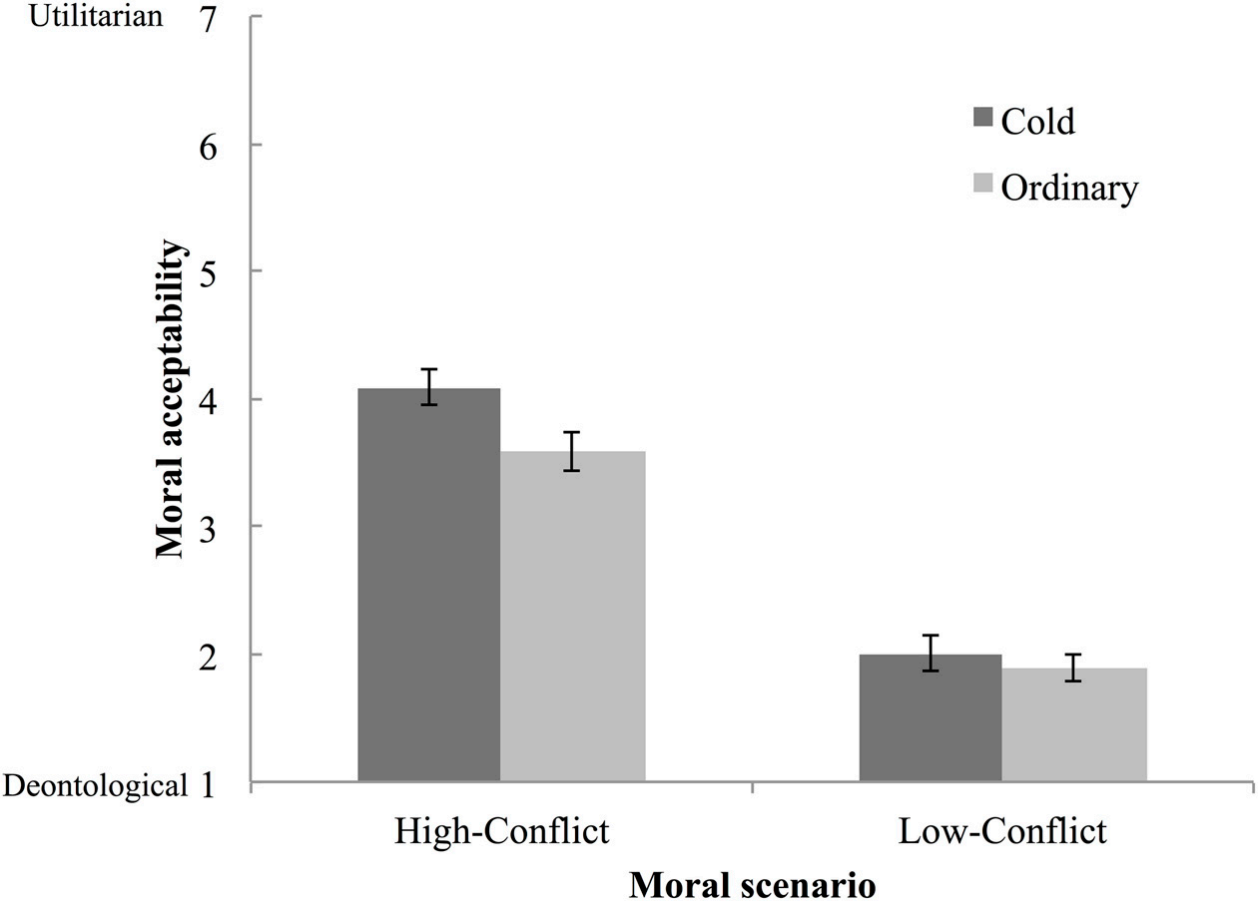
**Mean moral acceptability ratings and 95% CI (Cousineau-Morey-Baguley's Difference-Adjusted Normalized Confidence Intervals) as functions of temperature and moral scenario**.

The mean ratings of moral acceptability for each type of dilemma for each participant were analyzed (Figure 1). A Two-by-Two analysis of variance (ANOVA) was conducted. Temperature (cold, ordinary) was the between-participants factor, and moral scenario (high conflict, low conflict) was the within-participants factor. We also calculated Cousineau-Morey-Baguley's difference-adjusted normalized confidence intervals. There was a significant effect of moral scenario *F*_(1, 44)_ = 360.89, *p* < 0.001, η^2^_*g*_ = 0.69, a marginally significant effect of temperature *F*_(1, 44)_ = 3.81, *p* = 0.057, η^2^_*g*_ = 0.06, and an interaction between temperature and scenario *F*_(1, 44)_ = 3.67, *p* = 0.062, η^2^_*g*_ = 0.02. A simple main effect of temperature was significant in the high-conflict scenario *F*_(1, 44)_ = 5.91, *p* = 0.019, η^2^_*g*_ = 0.12, but it was not significant in the low-conflict scenario *F*_(1, 44)_ = 0.54, *p* = 0.464, η^2^_*g*_ = 0.01. The participants made more utilitarian judgments in the cold temperature condition than in the ordinary temperature condition for high-conflict moral dilemmas, *M*_cold_ = 4.09 [95% *CI*: 3.95, 4.24], *M*_ordinary_ = 3.59 [3.44, 3.74], but not for low-conflict moral dilemmas, *M*_cold_ = 2.02 [1.88, 2.17], *M*_ordinary_ = 1.90 [1.79, 2.04]. As in previous studies (Koenigs et al., 2007; Moretto et al., 2010), deontological judgments were dominant in the low-conflict personal moral dilemma scenarios, regardless of temperature manipulation; therefore, subsequent analysis focused on the results of high-conflict personal moral dilemmas.

#### Construal level and RTs

**Figure 2:**
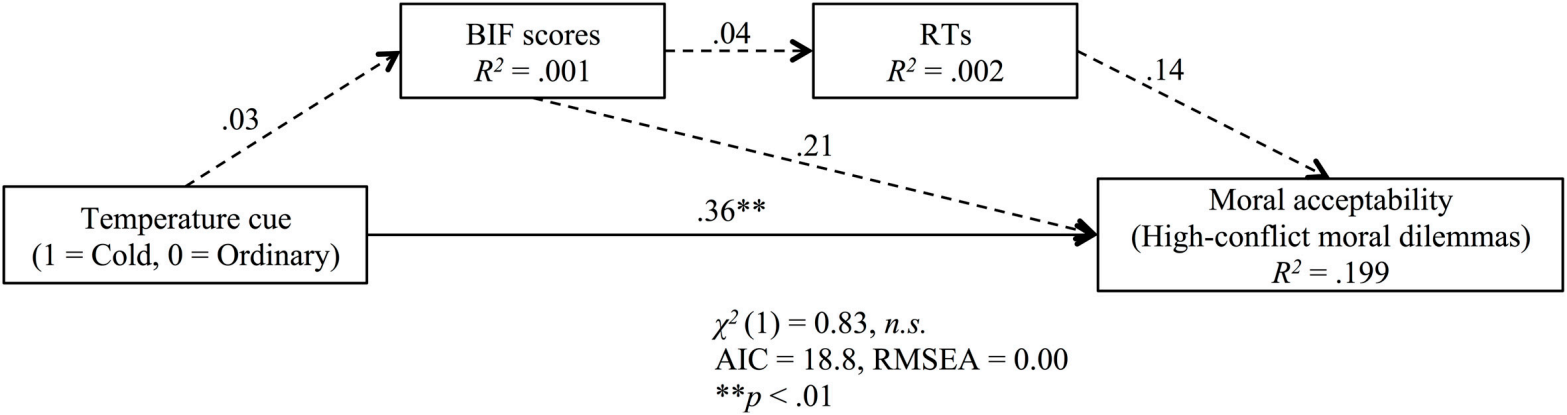
**Standardized coefficients for the relationship between temperature cue and moral acceptability as mediated by construal level (BIF scores) and RTs**. All paths are included in the model. Solid line indicated *p* < 0.10, and dashed line indicated *p* ≥ 0.10.

Path analysis was used to test whether the effect of coldness on high-conflict personal moral dilemma was mediated by construal level and RTs (Figure 2). The results indicated coldness did not have a significant effect on BIF β = 0.03, *p* = 0.862, and there were no significant effects of BIF scores on RTs β = 0.04, *p* = 0.784 and moral dilemma judgment β = 0.21, *p* = 0.110. Furthermore, there was no significant effect of RTs on high-conflict personal moral dilemma judgments β = 0.14, *p* = 0.283. Coldness directly facilitated the acceptability of utilitarian judgment β = 0.36, *p* = 0.008.

We also conducted a multiple regression analysis to test the differences between the cold and room temperature conditions' in effect of construal level and the RTs on moral judgment. In the ordinary room temperature condition, construal level and RTs predicted 16% of the variance, *adjR*^2^ = 0.16, *F*_(2, 20)_ = 3.08, *p* = 0.068, and BIF score significantly predicted acceptance of a utilitarian judgment, β = 0.49, *p* = 0.022, *VIF* = 1.02, while RTs did not, β = −0.09, *p* = 0.648, *VIF* = 1.02. On the other hand, in the cold temperature condition, construal level and RTs did not significantly explain the variance, *adjR*^2^ = 0.005, *F*_(2, 20)_ = 1.05, *p* = 0.368, and neither BIF score, β = 0.04, *p* = 0.850, *VIF* = 1.00, nor RTs, β = 0.31, *p* = 0.164, *VIF* = 1.00, predicted moral acceptability.

#### Affective state

Analysis of the PANAS scores showed that there were no statistically significant effects of temperature cue on the positive-negative affective state at the start of the experiment [positive *t*_(44)_ = 0.02, *p* = 0.980, *d* = 0.01; negative *t*_(44)_ = 0.32, *p* = 0.749, *d* = 0.10], or after the physical temperature was manipulated [positive *t*_(44)_ = 0.07, *p* = 0.945, *d* = 0.02; negative *t*_(44)_ = 0.71, *p* = 0.481, *d* = 0.21], or after the moral judgment task [*t*_(44)_ = 0.42, *p* = 0.676, *d* = 0.12; negative *t*_(44)_ = 0.43, *p* = 0.669, *d* = 0.13]. There were no significant correlations between acceptability of a utilitarian decision in high-conflict moral dilemmas and positive or negative affective state before the moral judgment was made (2nd round of PANAS): *r*_positive_ = 0.10, *p* = 0.496, *r*_negative_ = 0.07, *p* = 0.655.

### Discussion

We found that physical coldness facilitated utilitarian moral judgment in high-conflict personal moral dilemmas, but the cold cue had no significant effect on affective state, construal level, or moral judgment latency. A high-level construal mindset was related to utilitarian judgment in only the ordinary room temperature condition, while there was no significant relationship between high-level construal and RTs. These results imply that physical coldness and high-level construal did not prompt deliberate thought; rather, they might decrease the empathic concern and emotional aversion to sacrificing.

The effect of the cold temperature, which was independent from that of construal level and RTs, implicated that coldness facilitated utilitarian judgment via reducing empathic concerns. Warmth is related to communal sharing relationships and unity moral motives, which link empathy and compassion (IJzerman and Koole, 2011; Rai and Fiske, 2011), and lack of empathic concern directs utilitarian moral judgment without engaging deliberate processes (Wiech et al., 2013). Participants in the cold condition could easily make a utilitarian moral judgment because they did not need to evoke the deliberate process in order to suppress an emotional response. In addition, even though the low-level construal mindset focused on concrete means, it might be hard to trigger an empathic deontological response in the cold condition. Therefore, coldness prompted utilitarian judgment, regardless of RTs and construal level.

In the room temperature condition, high-level construal mindset predicted utilitarian judgment, while no effect of RTs on moral dilemma judgment was observed. Eyal et al. (2008) posited that the distinction between moral judgments based on high- and low-level construal is not related to the distinction between effortless intuition-based judgment and the reflective reasoning process. Participants in the high-level construal mindset might pay more attention to abstract utilitarian features rather than concrete deontological features; thus, the emotional-deontological response was not strongly triggered. Consequently, the conflict between deontological and utilitarian judgments might be low in the high-level construal mindset; therefore, the high-level construal could direct utilitarian judgment without engaging deliberate processes.

Contrary to our prediction, we did not observe any effect of coldness on construal level. There may be individual differences in the relationship between temperature perception and psychological distance other than social distance. For example, cold temperature is temporally closer than a warmer temperature in the winter season; therefore, it is possible that even though a cold cue increased social distance, it is not associated with other psychological distance dimensions.

In summary, the results of Experiment 1 showed that coldness facilitates utilitarian judgment regardless of affective states, deliberate thought, and abstract high-level construal mindset. In Experiment 2, we examined whether coldness puts people in a “*cold-hearted*” state. We tested the effect of coldness on social distance and empathic concern for victims potentially sacrificed in a moral dilemma judgment.

## Experiment 2

Experiment 2 tested whether coldness affects moral dilemma judgment via reducing empathic concern for potentially sacrificed victims. Temperature perception affects social distance, and it is related to unity moral motives, which link empathy and compassion (IJzerman and Semin, 2009; Rai and Fiske, 2011). Furthermore, reduction of empathic concern and emotional aversion to harming others were related to an increased utilitarian moral judgment (e.g., Gleichgerrcht and Young, 2013; Wiech et al., 2013). We hypothesized that physical coldness may increase social distance from the sacrificed victims and may decrease empathic feelings, thus facilitating utilitarian decisions in high-conflict personal moral dilemma judgments. In addition, we examined whether coldness directly decreased empathic feelings. Even though the relationship between temperature and empathy stems from social proximity, it is possible that the temperature cue may directly activate empathy-related concepts, such as “warm-hearted” or “cold-hearted,” and it may affect empathic feelings.

### Methods

#### Participants

This study was approved by the faculty ethics committee, and all the participants provided informed consent. Forty-one Japanese undergraduates (*M*_age_ = 19.73, *SD*_age_ = 1.12; 17 female, 24 male) participated in Experiment 2 in exchange for a partial course credit.

#### Measures

***Moral judgment task***. The target stimuli in the present study were eight high-conflict personal moral dilemmas, and we added eight low-conflict personal moral dilemmas to reduce repetition. The high-conflict moral dilemma scenarios: were Crying Baby, Footbridge, Sophie's Choice, Vitamins, Lawrence of Arabia, Submarine, Bomb, and Preventing the Spread. The low-conflict personal moral dilemmas were the same as in Experiment 1.

***Inclusion of other in the self-scale***. To measure the social distance from the sacrificed individuals, we asked the participants to remember the moral dilemma scenarios as a whole and then rate the overlap between themselves and the people who were going to be sacrificed in the moral dilemma scenarios on the 7-point Inclusion of Other in the Self (IOS) scale (Aron et al., 1992). The IOS measures the level at which “the other is included in the self,” and this scale presented seven diagrams of two circles varying in their degree of overlap. The overlap between the circles represented inclusion and social distance (lower scores indicated greater social distance).

***Empathy scale***. To test the empathic feelings during the moral dilemma judgment, we presented four empathy-related adjectives: *sympathetic, compassionate, softhearted*, and *tender* (Zhou et al., 2012). The participants were asked to rate the extent to which they had experienced the four emotional states while answering the moral dilemma task on a 7-point scale (1 = *not at all*, 7 = *very much*).

#### Procedure

This procedure was almost the same as in Experiment 1. This experiment was presented as two unrelated studies. One was ostensibly a “commercial product evaluation” study, and the second was a “judgment” study. First, the participants were asked to participate in a “commercial product evaluation” study and were given a scarf with either frozen internal water packs or internal water packs at room temperature. They were instructed to put the scarf around their necks and answer 7 questions about the scarf (e.g., “This scarf is the right size”) on a 7-point scale (1 = *Strongly disagree*, 7 = *Strongly agree*). At this point, to remove any possible interfering effects from the associative network of temperature-related words, we did not ask questions about temperature before the moral judgment task. After the product evaluation questions, the participants answered the PANAS. Then, they were told that this product evaluation study also examined the prolonged use of the scarf and that they were to keep wearing the scarf. Then, the experimenter asked to join a judgment study that was not related to the product evaluation study. In this study, the participants responded to 16 moral dilemma scenarios. Following the moral dilemma task, the participants completed the IOS scale and an empathy rating. Participants then responded to the product evaluation questions again and answered 10 questions about the scarf. The second round product evaluation section included one question about product temperature: “*This scarf is cold.*” The final task was to complete the second round of the PANAS. After the experiment, the participants were thanked and debriefed. None of them reported being suspicious about the experimental purpose. Participants took about 40 min to complete the all tasks.

### Results

Table 2 shows the means and 95% confidence intervals of temperature, coldness rating, IOS scale, Empathy scale, PANAS scores, and RTs both in the cold and the ordinary room temperature conditions. RTs were natural log transformed before analysis, and mean RTs were obtained via inverse transformation.

**Table 2:** **Means and 95% CIs of temperature, coldness rating, moral acceptability, IOS scale, Empathy scale, PANAS scores, and RTs in cold and ordinary room temperature conditions in Experiment 2**.

		**Cold**			**Ordinary**		
**Measures**		**Mean**	**95% CI**		**Mean**	**95% CI**	
Room temperature	(Celsius)	27.43	27.03	27.83	27.50	27.22	27.78
Coldness rating		6.38	5.96	6.80	2.80	2.11	3.49
Moral acceptability	Low conflict	1.90	1.71	2.09	1.74	1.62	1.87
	High conflict	3.97	3.78	4.16	3.40	3.27	3.53
RTs (ms)	Low conflict	4092			3881		
	High conflict	5382			5152		
IOS scale		3.33	2.45	4.21	3.85	2.98	4.71
Empathy scale		3.24	2.80	3.68	3.79	3.28	4.29
PANAS 1st	Positive	2.44	2.08	2.81	2.43	2.09	2.77
	Negative	2.03	1.73	2.33	2.19	1.94	2.43
PANAS 2nd	Positive	2.14	1.78	2.51	2.27	1.91	2.63
	Negative	2.32	1.86	2.77	2.20	1.84	2.56

#### Manipulation check

There was no significant difference in room temperature between the cold and ordinary room temperature conditions, *t*_(39)_ = 0.30, *p* = 0.763, *d* = 0.10. The scarf with the frozen internal water packs was evaluated to be colder than the scarf with internal water packs at room temperature, *t*_(39)_ = 9.39, *p* < 0.001, *d* = 3.01.

#### Moral judgment

The mean ratings of moral acceptability for each type of dilemma for each participant were analyzed. A Two-by-Two ANOVA was conducted. Temperature (cold, ordinary) was the between-participants factor and moral scenario (high conflict, low conflict) was the within-participants factor. The main effects of moral scenario, *F*_(1, 39)_ = 285.86, *p* < 0.001, η^2^_*g*_ = 0.71, and temperature, *F*_(1, 39)_ = 5.33, *p* = 0.026, η^2^_*g*_ = 0.08, were significant, and the interaction between temperature and scenario was marginally significant, *F*_(1, 39)_ = 3.54, *p* = 0.067, η^2^_*g*_ = 0.03. A simple main effect of temperature was significant in the high-conflict scenario *F*_(1, 39)_ = 6.45, *p* = 0.015, η^2^_*g*_ = 0.14, while effect of temperature was not significant in the low-conflict scenario, *F*_(1, 39)_ = 1.03, *p* = 0.315, η^2^_*g*_ = 0.03. Participants made more utilitarian judgments in the cold temperature condition than in the room temperature condition for high-conflict moral dilemmas. As in Experiment 1, further analysis was conducted on the high-conflict dilemmas.

#### Social distance and empathy

**Figure 3:**
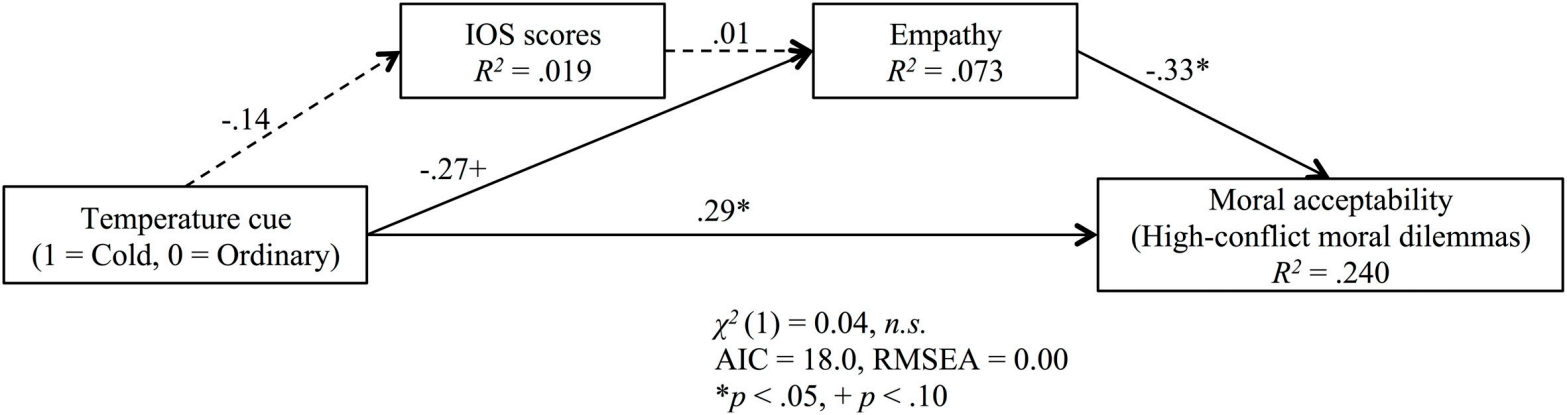
**Standardized coefficients for the relationship between temperature cue and moral acceptability as mediated by social distance (IOS scores) and empathy**. All paths are included in the model. Solid line indicated *p* < 0.10, and dashed line indicated *p* ≥ 0.10.

Path analysis was used to test whether the effect of coldness on high-conflict personal moral dilemma judgment was mediated by social distance toward sacrificing victims and empathic feelings (Figure 3). The results indicated that coldness had a marginally significant effect on empathy β = −0.27, *p* = 0.082, and coldness positively affected the acceptability of utilitarian decisions β = 0.29, *p* = 0.045. Empathy negatively affected the acceptability of utilitarian decisions β = −0.33, *p* = 0.022, while coldness did not affect social distance β = −0.14, *p* = 0.375, and social distance did not affect empathy β = 0.01, *p* = 0.930. To examine whether empathy mediated the effect of coldness on moral acceptability, we conducted a bootstrapping analysis with 1,000 bootstrapped samples. The indirect effect between coldness and moral acceptability as mediated through empathy was marginally significant: β = 0.09 in a point estimate, *SE* = 0.07, and a 95% *CI* was −0.01 to 0.269, and a 90% *CI* was 0.001 to 0.232. We also conducted bootstrapping analysis to test a total effect of coldness on moral acceptability. The total effect of coldness on moral acceptability was significant: β = 0.38 in a point estimate, *SE* = 0.12 and 95% *CI* was 0.108 to 0.579.

#### RTs

A Two-by-Two ANOVA was conducted for RTs. Temperature (cold, ordinary) was the between-participants factor, and moral scenario (high conflict, low conflict) was the within-participants factor. The main effects of moral scenario were significant, *F*_(1, 39)_ = 44.84, *p* < 0.001, η^2^_*g*_ = 0.11, while the effect of temperature, *F*_(1, 39)_ = 0.17, *p* = 0.676, η^2^_*g*_ = 0.004, and the interaction between temperature and scenario were not significant, *F*_(1, 39)_ = 0.01, *p* = 0.912, η^2^_*g*_ < 0.001. The RTs of the low-conflict moral dilemmas were faster than those of the high-conflict moral dilemmas. There were no significant correlations between acceptability of utilitarian decision in moral dilemmas and RTs: *r*_low−conflict_ = 0.09, *p* = 0.582, *r*_high−conflict_ = −0.07, *p* = 0.683.

#### Affective state

Analysis of the PANAS scores showed that there were no statistically significant effects of temperature cues on the positive-negative affective state after physical temperature was manipulated [positive *t*_(39)_ = 0.04, *p* = 0.970, *d* = 0.012; negative *t*_(39)_ = 0.85, *p* = 0.403, *d* = 0.21], or after the moral judgment task [positive *t*_(39)_ = 0.51, *p* = 0.611, *d* = 0.16; negative *t*_(39)_ = 0.61, *p* = 0.543, *d* = 0.27]. There were no significant correlations between the acceptability of utilitarian decisions in high-conflict moral dilemmas and positive or negative affective state before the moral judgment (1st round of PANAS): *r*_positive_ = 0.10, *p* = 0.548, *r*_negative_ = −0.12, *p* = 0.468.

### Discussion

The results of Experiment 2 partially support the hypothesis that coldness facilitated utilitarian judgment through reducing empathy. What is more, the direct effect of coldness on moral dilemma judgments was also significant. Contrary to our prediction, there was no significant effect of coldness on social distance, and effect of social distance on empathic feelings was not significant. As in Experiment 1, effects of coldness on judgment latency and affective states were not observed.

Previous studies showed that lack of empathy and emotional aversion to harming others prompted utilitarian judgment (e.g., Gleichgerrcht and Young, 2013; Wiech et al., 2013). Empathy is connected with unity moral motives, which motivates individuals to take care of people in communal sharing relationships, such as families and close friendships (Rai and Fiske, 2011). Communal sharing relationships are made up of bonding experiences, including bodily touching (IJzerman and Koole, 2011). The results of Experiment 2 were compatible with these views: coldness reduced empathic feelings, and reduction of empathic feelings prompted utilitarian judgment. However the effect of coldness on empathy did not reach statistically significant effect (β = −0.27, *p* = 0.082). One possible explanation is that this experiment presented an explicit questionnaire to measure empathy. The emotional processes in moral dilemma judgment are fast and automatic (Greene et al., 2004, 2008), the relationship between temperature and social cognition is said to be very basic (IJzerman and Koole, 2011), and studies of embodied cognition indicate that participants were not aware of the effect of bodily state (Barsalou, 2008; Williams and Bargh, 2008). Together with this, it is possible that coldness prompts utilitarian judgment via unconscious emotional processes, and participants' subjective report of their empathic state could not fully reflect this unconscious process. The direct effect of coldness on moral dilemma judgment might reflect such unconscious effects of physical temperature on moral dilemma judgment.

We did not observe an effect of coldness on social distance toward the sacrificed victims or an effect of social distance on empathy. Therefore, coldness might directly activate empathy-related concepts (e.g., cold-hearted) and reduce empathic concerns. Note that the present experiment asked individuals to evaluate the overlap between themselves and the victims as a whole, and this effect might be different from participants to participants, in which case the victim is more vividly remembered (e.g., a man on the footbridge or a crying baby). Consequently, it is possible that the results of the IOS scale did not reflect the social distance toward victims as a whole; rather, it reflected the differences in social distance toward the vividly remembered victim. In addition, present experiment did not measure social distance toward saved people, and did not separately ask empathic concern toward the sacrificing victim and toward the saved people. Previous studies of temperature and social cognition presented neutral person, not a suffering person, as a target (Williams and Bargh, 2008), therefore coldness might affect social distance and empathic concern toward not only sacrificing victims but also saved people. It is plausible that coldness increased social distance toward all people in the dilemma scenario and decreased empathic feelings during the judgment, and then prompted utility-based judgment. Further research will be needed to test whether the effect of temperature on empathy is mediated by social proximity, and whether the coldness affects social proximity and empathy toward the sacrificing victim or all people in the scenario.

## General discussion

The present experiments provide preliminary evidence that physical coldness prompted utilitarian moral decisions by making people more “cold-hearted”; rather than “cool-headed.” There are three ways to facilitate utilitarian judgment. The first is to engage in deliberate thought to suppress an immediate emotional response (Greene et al., 2008). The second is to focus on abstract goals (Aguilar et al., 2013), and the third is to reduce empathic concern and decrease aversion to sacrificing others (Wiech et al., 2013). The results of Experiment 1 indicate that the effect of coldness on moral dilemma judgment was independent from “cool-headed,” deliberate thought or from the abstract, high-level construal mindset. Experiment 2 suggests that the effect of coldness on moral dilemma judgment was partially mediated by a reduction of empathic feelings. Previous studies have pointed out that individuals higher on psychoticism or psychopathy scales, which are characterized by a lack of empathic concern, showed utilitarian judgment without engaging in rational-deliberate processing (Wiech et al., 2013). Therefore, an extremely cold temperature led to people being labeled as “cold-hearted.” They exhibited a less empathic state and more easily accepted utilitarian decisions.

Studies of moral dilemma judgment showed scenarios that involved physical contact or personal force elicited fewer utilitarian decisions (Cushman et al., 2006; Greene et al., 2009). Our present experiments reveal that embodiment actually affects moral dilemma judgment, and the results imply that temperature perception underlie social relationships and morality. Intimate social relationships such as communal sharing relationships are scaffolded onto physical experiences like touching others and sensing others' temperature (IJzerman and Koole, 2011; Rai and Fiske, 2011), and thus temperature perception may cue communal sharing relationships and unity moral motives, which directs to take care in-group member and activate empathy and compassion. Present experiments support these views: coldness decreased empathic feeling and increase utilitarian moral decisions. Thus, temperature perception or physical contact may activate concepts of close relationship with others, and directed moral emotions and judgments.

Future research needs to focus on several issues that still remain unanswered. The first is the relationship between temperature, social distance, and moral judgment. It needs to be determined whether coldness increases the social distance toward the sacrificed victims, or if just activates the concept related to coldness. The second issue is the bidirectional feature of temperature perception and social cognition (Zhong and Leonardelli, 2008; IJzerman and Semin, 2010). Do utilitarian decisions induce people to perceive physical coldness? The third issue is more automatic and involves the unconscious effect of temperature on moral dilemma judgment. Moretto et al., (2010) showed that patients with emotion-related brain regions (e.g., vmPFC) made more utilitarian judgment and failed to generate a SCR before making utilitarian judgment. Wiech et al. (2013) indicated that participants with antisocial psychoticism traits showed less activation of the subgenual cingulate cortex, which is related to empathic concerns, during their utilitarian judgment making. Therefore, further research is needed to test whether temperature affects the automatic emotional response during moral dilemma judgment by measuring SCRs or brain activation.

In conclusion, it is important to be aware that our moral judgment and empathic feelings are affected by temperature perception. Gockel et al. (2014) revealed that ambient temperature affects the judgment of criminals: participants in a lower temperature room regarded criminals as more cold-blooded and ascribed higher degrees of penalties to them. Similarly, present results imply that coldness affects a jury's empathic concern toward a victim, thereby affecting a verdict. The present study bridges embodied cognition and moral judgment and implies that our morality is scaffolded onto the our bodily experiences.

## Author contributions

All authors contributed to the study concept development and study design under the supervision of Jun Kawaguchi. Hiroko Nakamura, Yuichi Ito, Yoshiko Honma, and Takuya Mori constructed the experimental materials. Takuya Mori and Hiroko Nakamura performed the testing and data collection. Hiroko Nakamura and Yuichi Ito performed the data analysis and interpretation with additional input from all the authors. Hiroko Nakamura drafted the manuscript, and Yuichi Ito and Jun Kawaguchi provided critical revisions. All authors approved the final version of the manuscript for submission.

### Conflict of interest statement

The authors declare that the research was conducted in the absence of any commercial or financial relationships that could be construed as a potential conflict of interest.

## Supplementary material

Datasheet of Experiments 1 and 2 are available at Supplementary materials with excel file.

Table and forest plot about the meta-analysis of mean differences between cold condition and ordinary condition in moral acceptability of high-conflict moral dilemma is available at Supplementary materials. *Post-hoc* analysis revealed statistical power of the difference between cold and ordinary temperature condition in moral acceptability of high-conflict moral dilemma was 0.83 in Experiment 1, and 0.78 in Experiment 2 (one-tailed). To increase statistical power, we conducted meta-analysis of Experiments 1 and 2 (Cohn and Becker, 2003). The results showed that temperature manipulation affected acceptability of high conflict moral dilemma *d*_unbiased_ = 0.78 [0.346, 1.203].
