# Supplementary material for: Cold-hearted or cool-headed: physical coldness promotes utilitarian moral judgment
Source: Front Psychol. 2014 Oct 2;5:1086. doi: 10.3389/fpsyg.2014.01086 (PMC4183093; doi:10.3389/fpsyg.2014.01086)

Meta analysis of mean differences between cold condition and ordinary condition in moral acceptability of high-conflict moral dilemma

Study	N	$d_{unbiased}$	V	95% CI for δ	
Experiment 1	46	0.783	0.091	0.191	1.394
Experiment 2	41	0.765	0.101	0.139	1.412
Combined		0.775	0.048	0.346	1.203

Heterogeneity $Q = 0.002$, $df = 1$, *n.s.*, $I^2 = 0.0\%$

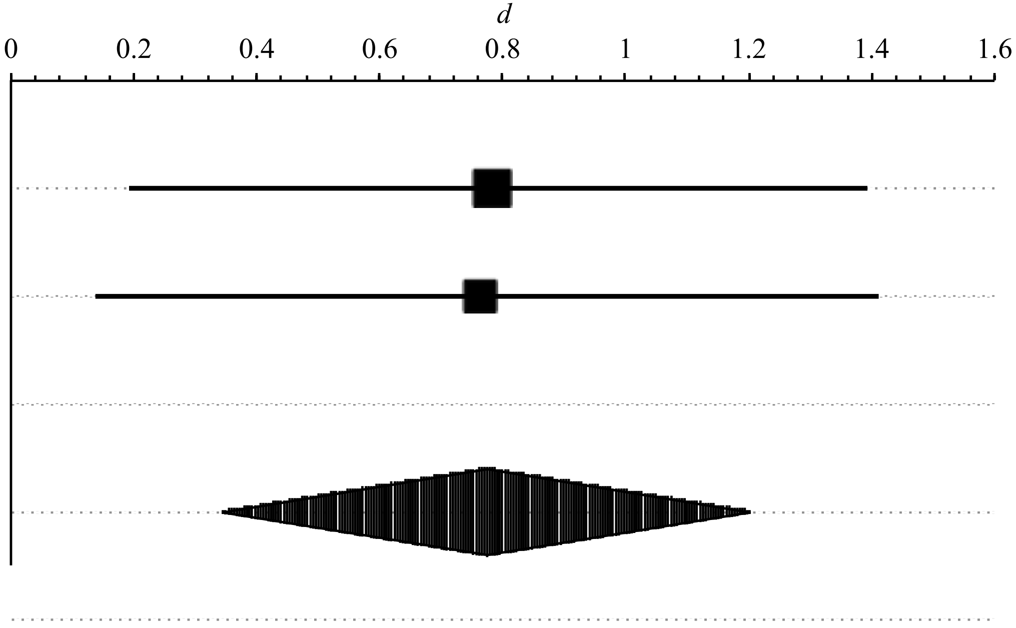

Supplement: Supplementary file 1 [file Table1.PDF]
